# Identity Resilience, Community Connectedness, and Sociosexuality Among Gay and Bisexual Men: The Mediating Effect of Internalized Homonegativity

**DOI:** 10.3390/ijerph23030358

**Published:** 2026-03-12

**Authors:** Anthony J. Gifford, Rusi Jaspal

**Affiliations:** 1 Department of Psychology & Counselling, School of Life and Health Sciences, Birmingham City University, Birmingham B4 7BD, UK; 2 Vice Chancellor’s Office, University of Brighton, Brighton BN2 4AT, UK; r.jaspal@brighton.ac.uk

**Keywords:** identity resilience, community connectedness, internalized homonegativity, sociosexuality, gay and bisexual men

## Abstract

**Highlights:**

**Public health relevance—How does this work relate to a public health issue?**

- Sociosexuality among gay and bisexual men has been framed within public health primarily as a precipitant of sexual risk, particularly in relation to HIV and other sexually transmitted infections. This study reframes that discourse by examining sociosexuality alongside aspects of sexual health promotion, such as PrEP and condom use self-efficacy.
- By situating sociosexuality within identity processes, community connectedness, and internalized homonegativity, this work addresses broader public health concerns relating to mental health, wellbeing, and health-promoting sexual decision-making among sexual minority men.

**Public health significance—Why is this work of significance to public health?**

- The findings challenge deficit-based and risk-only public health narratives by demonstrating that sociosexuality is also associated with positive identity profiles and psychosocial wellbeing, rather than solely reflecting pathology or poor risk appraisal.
- By showing that internalized homonegativity mediates associations between identity resources and sociosexuality, the study identifies internalized homonegativity as a modifiable identity evaluation construct with implications for both sexual health and mental health outcomes.

**Public health implications—What are the key implications or messages for practitioners, policy makers and/or researchers in public health?**

- Public health interventions and sexual health services should adopt sex-positive, stigma-informed approaches that recognize sociosexuality as a normative and potentially beneficial aspect of gay and bisexual men’s lives, particularly when supported by effective sexual health promotion strategies.
- Policies and programs that strengthen identity resilience and LGBT+ community connectedness, while actively addressing internalized homonegativity, may enhance both sexual wellbeing and engagement with HIV/STI prevention, demonstrating the value of integrated psychosocial and biomedical approaches in public health practice.

**Abstract:**

Sociosexuality refers to the proclivity to engage in casual sex without commitment and is generally operationalized in terms of attitudes, behavior, and desire. Moving beyond the dominant focus on sexual risk and pathology in studies of sociosexuality, this study conceptualizes sociosexuality as a positive psychological variable that reflects the enactment of sexual identity among gay and bisexual men. Using cross-sectional correlational survey data from 512 gay and bisexual men in the United Kingdom, the direct associations between sociosexuality and identity resilience and LGBT+ community connectedness, and indirect associations through the mediation of internalized homonegativity, were examined. Results showed that identity resilience was indirectly associated with higher sociosexuality via decreased internalized homonegativity, and that LGBT+ community connectedness was directly and positively associated with sociosexuality and indirectly via decreased internalized homonegativity. The findings suggest that the adaptive self-schema of identity resilience and the adaptive relational schema of community connectedness may militate against internalized homonegativity, which in turn may facilitate sociosexuality among gay and bisexual men. Interventions to support sexual identity enactment should therefore focus on developing these adaptive schemas.

## 1. Introduction

Sociosexuality refers to the proclivity to engage in casual sex without commitment and is generally operationalized in terms of attitudes (i.e., toward sex without commitment), behavior (i.e., number of sexual partners), and desire (i.e., degree of sexual arousal from casual partners) in relation to casual sex [1,2,3]. Empirical research generally shows higher levels of sociosexuality in men than in women [4,5], and in gay and bisexual men compared to heterosexual men [2,3,6,7].

Many studies of gay and bisexual men have examined sociosexuality through the lenses of pathology and risk, focusing particularly on sexual risk (namely vulnerability to sexually transmitted infections (STIs) and human immunodeficiency virus (HIV)), largely from the perspectives of health psychology and public health [2,8,9]. Yet sociosexuality reflects more than this—for many gay and bisexual men, it is a key component of forming meaningful relations and a personal community [10]. Moreover, in the era of effective antiretroviral therapy for both the treatment and prevention of HIV, pre-exposure prophylaxis (PrEP) and, more recently, doxycycline post-exposure prophylaxis (DoxyPEP) for the prevention of bacterial STIs [11,12,13], it is appropriate to examine sociosexuality in gay and bisexual men from a positive psychological perspective, that is, as a prosocial variable that reflects the enactment of one’s sexual identity, intimacy, pleasure, enjoyment, interpersonal connection, and sexual freedom [10,14,15,16].

This study examines the system of factors that are associated with sociosexuality in gay and bisexual men. Both the self-schema of identity resilience (that is, the extent to which one’s identity is characterized by self-esteem, self-efficacy, continuity, and positive distinctiveness) and the relational schema of community connectedness have been found to have affirmative and positive effects upon various dimensions of health and wellbeing in gay and bisexual men [17,18]. Conversely, internalized homonegativity is consistently found to be associated with poor health and wellbeing outcomes [19,20]. Accordingly, this study examines the direct associations between identity resilience and LGBT+ community connectedness and sociosexuality and indirect associations through the mediation of internalized homonegativity.

### 1.1. Psychological Underpinnings of Sociosexuality

Extant research tends to examine sociosexuality dichotomously as being either “restricted” (low levels) or “unrestricted” (high levels). Many studies have found “unrestricted” sociosexuality to be predicted by various maladaptive or pathological self-schemas, such as dark triad traits, aggression, anger/hostility, impulsivity, lack of constraint, lower agreeableness, insecure attachment styles, irresponsibility, and others [21,22,23,24]. Moreover, studies have shown higher levels of sexual guilt and sex-related anxiety in people with higher sociosexuality [23]. Sociosexuality has also been found to be associated with engagement in maladaptive behaviors, such as infidelity [25], the sexual harassment of others [26,27] and problematic pornography use [28]. These studies, focusing mainly on heterosexual male samples, tend to conceptualize sociosexuality as a negative cognitive, affective, and behavioral construct. Additionally, studies of sociosexuality in gay and bisexual men tend to examine its relationship with sexual risk, that is, the risk of exposure to STIs and HIV [2,29]. Clearly, sociosexuality is not invariably a positive psychological construct.

However, a deeper analysis of commentary and empirical research on gay and bisexual men reveals that engagement in casual sexual encounters with multiple partners may, at least for some, constitute a means of forming interpersonal connections which can develop into acquaintances, friendships, and other meaningful relationships. This is particularly important in view of the minority stressors (e.g., stigma, prejudice, discrimination) to which gay and bisexual men are subjected in heteronormative contexts, spurring the need for connection, belonging, and community [30]. Indeed, Wilkinson et al.’s nationwide study of 4000 gay and bisexual men showed that the vast majority of respondents reported developing friendships following casual sexual encounters, suggesting that sex-seeking is linked to sociability [10]. Similarly, others have suggested that sexuality (i.e., initial sexual encounters) constitutes an initial key component of gay and bisexual men’s friendships even if sexual interest wanes over time [31,32,33,34]. Furthermore, in their study of gay and bisexual men’s motivations for frequenting gay saunas, Jaspal and Papaloukas [14] found that casual sexual encounters in the sauna provided individuals with feelings of sexual identity, authenticity, and belongingness, and that it alleviated isolation, loneliness, and depression.

**Figure 1:**
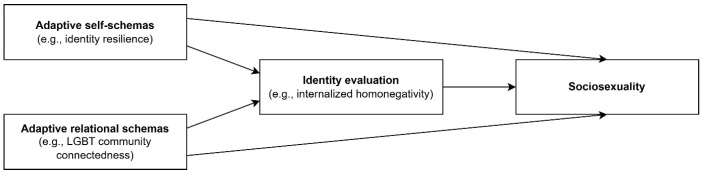
A theoretical model of adaptive relational and self-schemas, identity evaluation, and sociosexuality in gay and bisexual men.

As such, under some circumstances, sociosexuality can be thought of as a positive psychological construct. Moreover, in contrast to the binary examination of sociosexuality as either restricted or unrestricted, the present study operationalizes the construct as a continuous variable, focusing on the degree of sociosexuality. It is important to understand its underpinnings, that is, the system of psychological factors that may predict sociosexuality. This approach should elucidate some of the circumstances under which sociosexuality operates as a positive psychological construct. In this study, identity variables operating at the levels of self and other (i.e., self and relational schemas) are examined. More specifically, a theoretical model of adaptive relational and self-schemas, identity evaluation, and sociosexuality in gay and bisexual men (Figure 1) is tested.

The proposed model conceptualizes sociosexuality among gay and bisexual men as a potentially positive psychological construct that facilitates the affirmation and enactment of sexual identity and rejection of heteronormative sexual norms. As such, the model posits that the adaptive relational and self-schemas of LGBT+ community connectedness and identity resilience, respectively, should be directly and positively associated with sociosexuality and indirectly through the mediation of decreased homonegativity. In short, how people think about the self and the self in relation to others should influence the evaluation of their identity, which in turn should influence their degree of sociosexuality.

### 1.2. Sociosexuality and Sexual Health

While sociosexuality can, and should, be accepted as a normative and potentially beneficial facet of gay and bisexual men’s lives, it should be acknowledged that sex with multiple partners increases the risk of exposure to STIs, including HIV [34]. Moreover, when people are more depressed but engage in sociosexuality they may engage in more sexual risk behaviors [35]. Therefore, it should be acknowledged that sociosexuality is not invariably positive under all circumstances.

Nonetheless, purely risk-based approaches to sociosexuality overlook its social and psychological functions and tend not to promote affirmative understandings of sex between men [36]. Public health discourse has thus shifted from seeking an unrealistic ideal of “safe sex” toward promoting “safer sex” practises [37,38]. Safer sex practises should, therefore, not focus exclusively on the prevention of STIs/HIV but also incorporate pleasure, intimacy, and sexual satisfaction. Emerging research has begun to embed sexual pleasure as a legitimate component of sexual health and wellbeing, rather than focusing solely on “risk management” [39]. Sociosexuality should be viewed more broadly in terms of its implications for both sexual health and social, psychological, and relational wellbeing.

This is especially important in view of significant biomedical advances that have transformed the landscape of HIV and STI prevention. Interventions, such as PrEP, treatment as prevention (TasP; the principle that undetectable equals untransmittable [U = U]), and DoxyPEP have substantially reduced the biomedical risk historically associated with casual sex among gay and bisexual men [40]. These developments facilitate a model of sexual health that is compatible with, rather than opposed to, sociosexual expression. Accordingly, sociosexuality must be examined in light of these changing prevention contexts, which allow gay and bisexual men to pursue sexual connection and pleasure with greater scope to implement safer practices in ways that align with their own needs, preferences, and desires.

If sociosexuality is to be understood as a potentially positive dimension of wellbeing, it is necessary to examine the psychological conditions under which it can be safely and affirmatively enacted [41]. Thus, in addition to examining the roles of relational and self-schemas that in turn may shape how individuals experience and evaluate their sexual lives [42], this study also controls for the effects of two safer sex practices, namely condom use self-efficacy and PrEP use self-efficacy. Condom use self-efficacy refers to the individual’s general level of confidence to purchase, negotiate the use of, and actually use condoms in sexual encounters [43,44]. PrEP self-efficacy refers to “confidence in one’s ability to access and consistently take PrEP as prescribed” [45], p. 1. Rather than examining reported behavior, which is generally contingent upon context, we examine the general reported confidence in one’s own ability to engage in these significant safer sex behaviors.

### 1.3. Identity Resilience

In identity process theory, identity resilience is defined as an identity that is characterized by higher combined levels of self-esteem, self-efficacy, continuity, and positive distinctiveness [46]. According to the theory, these constitute the four principles that underpin a positive sense of identity. As such, identity resilience is conceptually distinguishable from the concept of psychological resilience, which generally focuses on self-efficacy and the individual’s ability to “bounce back” in the face of challenges [47]. Identity resilience develops over the individual’s life course and is shaped by many factors, including personality traits, social experiences, group memberships, and so on [48]. Although identity resilience is conceptualized as a relatively stable self-schema, it is possible to enhance identity resilience through therapeutic intervention [42].

Identity resilience is defined as the “capacity of the identity to resist its own invalidation, devaluation or fragmentation” [46], p. 581 in the face of challenges. As such, it is an adaptive self-schema that reflects the individual’s own subjective belief in their capacity to interpret and overcome challenges as they occur and their self-worth and self-value [48]. Empirical studies have found identity resilience to be protective against various forms of psychological disturbance, including body image concerns, psychological distress, anxiety, and depression [17,49,50]. Moreover, identity resilience is associated with greater satisfaction with life, relationships and, crucially, one’s sexuality [51,52].

People with higher identity resilience tend to have greater capacity to identify and adopt effective coping strategies when faced with challenges, such as the derivation of social support [53,54] and the adoption of health promotion behaviors [45]. Having a more resilient identity may promote a sense of self-worth and confidence to adopt coping strategies that may be risky in the short term but effective in the long term (e.g., self-disclosure, support-seeking, enacting one’s identity) [46]. Gay and bisexual men with higher identity resilience are more likely to come out to others, to derive community connectedness, and to have a more positive relationship with their sexuality [55]. They may feel more confident about asserting their identity in thought and deed. Therefore, we hypothesize that the adaptive self-schema of identity resilience will be associated directly and positively with sociosexuality (Hypothesis 1).

### 1.4. LGBT+ Community Connectedness

LGBT+ community connectedness is defined as the sense of identification with, and belonging to, a group with shared characteristics and that that group meets their social, psychological, and emotional needs [56]. Community connectedness is fundamentally concerned with the individual’s self-perception in relation to other members of their perceived community and, as such, can be conceptualized as an adaptive relational schema.

Sexual identity development models generally posit that engagement with the LGBT+ community can have empowering effects on both the development and enactment of one’s sexual identity [57,58,59]. For instance, gay and bisexual men who have greater community connectedness feel more comfortable about initiating and developing intimate relationships with other gay and bisexual men [59]. Community involvement may also provide opportunities to engage in casual sexual encounters, i.e., in sexual exploration [60].

Furthermore, as social identity theory [61] postulates, greater identification with the LGBT+ community is associated with greater endorsement of the norms, values, and social representations associated with that group. Indeed, casual sex with multiple partners is generally normalized in the gay and bisexual community, and traditional conceptions of romantic relationships (i.e., monogamy) are challenged with non-monogamy, “throuples”, and group sex being increasingly accepted and normalized [62]. As such, it is hypothesized that LGBT+ community connectedness will be associated directly and positively with sociosexuality (Hypothesis 2).

### 1.5. Internalized Homonegativity

Internalized homonegativity is defined as “the gay [or bisexual] person’s direction of negative social attitudes toward the self” [63], p. 161. These negative social attitudes shape the value that the individual appends to their sexuality, which reflects the evaluation process of identity construction [64,65]. As such, internalized homonegativity can be thought of as an identity evaluation variable. Internalized homonegativity is relatively common in gay men at earlier stages of sexual identity development due to exposure to negative social representations of homosexuality in largely heteronormative, and sometimes overtly homonegative, contexts [57,66]. Unlike identity resilience, it is not a stable self-schema and may reduce over time through exposure to more affirmative social representations of one’s sexuality [67], including through health and wellbeing interventions.

Relational and self-schemas, such as identity resilience and community connectedness, may reduce the risk of developing internalized homonegativity. For instance, in their experimental study of the psychological effects of recalling negative coming out experiences among gay and bisexual men, Breakwell and Jaspal found that higher identity resilience was associated with lower internalized homonegativity and less distress and identity threat upon recall of the negative coming out experience [50]. After all, a person with higher baseline self-esteem, self-efficacy, continuity, and positive distinctiveness may be better equipped to assert their sexual identity in the face of external distal stressors (e.g., stigma, prejudice, victimization) that may habitually engender internalized homonegativity.

On the other hand, sexual identity development models generally posit that engagement with the LGBT+ community is a key step toward reducing the internalized homonegativity experienced during the early stages of sexual identity development [57,58,59]. It is generally argued that community connectedness can provide exposure to more positive and affirmative social representations of homosexuality that compete with the negative social representations to which the gay or bisexual person is generally exposed in heteronormative contexts. Indeed, Petruzzella et al. found that greater community connectedness was associated with decreased internalizing symptoms, which would also include internalized homonegativity [68]. Furthermore, Jaspal found that LGBT+ community connectedness was associated with decreased sexual identity threat (that is, the perception that one’s sexuality undermines self-esteem, self-efficacy, continuity, and distinctiveness) [69].

When homonegativity is internalized, the gay or bisexual person experiences a range of negative social, psychological, and health outcomes [19,20]. Although there are no published studies of the association between internalized homonegativity and sociosexuality, studies have examined the effects of internalized homonegativity upon the related construct of sexual satisfaction. In their study of 110 older gay and bisexual men, Gonçalves et al. found that self-stigma (which amounts to internalized homonegativity) was negatively associated with sexual satisfaction [70]. Zheng and Zheng similarly found internalized homonegativity to be predictive of lower sexual satisfaction in their study of 403 Chinese gay and bisexual men [71]. Moreover, in their study of 199 gay men in the United Kingdom and Germany, Jaspal et al. found internalized homonegativity to be directly and negatively associated with sexual satisfaction [52]. Since the negative self-schema of internalized homonegativity is associated with feelings of self-disgust, self-hatred, and shame and secrecy due to one’s sexual orientation [20], gay and bisexual men with higher internalized homonegativity may be less inclined to enact their sexuality in ways that are indicative of sociosexuality.

Based on extant evidence, it is hypothesized that internalized homonegativity will mediate the associations between the adaptive schemas of identity resilience and LGBT+ community connectedness and sociosexuality. More specifically, both higher identity resilience and LGBT+ community connectedness should be associated with lower internalized homonegativity, which in turn should be associated with higher sociosexuality (Hypothesis 3).

### 1.6. The Current Study

The present study examines sociosexuality in gay and bisexual men as a potentially positive dimension of sexual and psychological wellbeing shaped by both identity processes and contemporary sexual health contexts. Drawing on identity process theory and related work on adaptive relational and self-schemas, we investigate whether identity resilience and LGBT+ community connectedness are associated with sociosexuality directly and indirectly via internalized homonegativity. In doing so, we conceptualize sociosexuality not as a marker of pathology or risk, but as a possible expression of identity affirmation, connection, and sexual agency. In line with recent biomedical advances that have transformed HIV and STI prevention, PrEP use self-efficacy and condom use self-efficacy are included as covariates to ensure that associations between identity-based factors and sociosexuality are examined independently of individuals’ confidence in engaging in safer sexual practices.

## 2. Materials and Methods

### 2.1. Design, Participants, and Procedure

Gay and bisexual men were recruited to take part in an online cross-sectional survey study between June–September 2023. Participants were eligible if they were aged 18 or over, were assigned male at birth, lived in the UK, self-identified as gay or bisexual men, and were not undergoing any gender-affirming care. The decision to limit the study to cisgender gay and bisexual men was based on the high likelihood that transgender and non-binary gay and bisexual people face unique stressors that may operate as barriers to sociosexuality, such as discrimination based on their gender and body image concerns [72,73], which may in turn operate as potential barriers to sociosexuality. These require careful examination with a suitable sample of gender-diverse gay and bisexual people in future research.

Participants were recruited using opportunistic sampling through social media (e.g., Twitter/X; San Francisco, CA, Instagram; Menlo Park, CA, Facebook; Menlo Park, CA). A total of 903 participants consented to complete the survey. Of these, N = 391 failed to complete the survey or failed the “bot-checker” built into the online survey platform [74]. A participant group of N = 512 was used for the final analysis. Participants aged between 18 and 73 years took part in the study (M = 35.61, SD = 9.95). Most participants (375; 73.2%) identified as gay, with the remainder identifying as bisexual. Additionally, 298 participants (58.2%) reported no history of PrEP use. In terms of ethnicity, 456 participants (89.1%) identified as White, 13 (2.5%) as Mixed, 17 (3.3%) as British Asian/Asian, 7 (1.4%) as Black, and 19 (3.7%) as Other. In terms of relationship status, 241 participants (47.1%) reported being single, while 269 (52.5%) reported being in a relationship, either monogamous or non-monogamous. There were no missing data from the final sample as these were removed prior to analysis. This study received a favorable ethical opinion from the Schools of Business, Law and Social Sciences Research Ethics Committee of Nottingham Trent University.

### 2.2. Measures

The Identity Resilience Index [48] is a 16-item scale providing an overall score of identity resilience (comprising self-esteem, self-efficacy, positive distinctiveness, and continuity) measured on a five-point Likert scale (1—strongly disagree, 5—strongly agree). Example items include “On the whole I am satisfied with myself” (self-esteem) and “I can always manage to solve difficult problems if I try hard enough” (self-efficacy). A higher mean score indicates higher identity resilience. This scale has shown excellent internal reliability in related research (α = 0.83 [75]) and in this study (α = 0.84).

The Revised Internalized Homophobia Scale [76] is a five-item scale measured using a five-point Likert scale (1—strongly disagree, 5—strongly agree). Wording was adapted to suit male participants. An example item is “I have tried to stop being attracted to men in general”. A higher mean score indicates higher internalized homonegativity. The scale has shown excellent internal reliability in previous studies (α = 0.88 [77]) and in this study (α = 0.83).

The Connectedness to the LGBT+ Community Scale [56] is an eight-item scale measured using a four-point Likert scale (1—strongly disagree, 4—strongly agree). An example item is “You feel a bond with the LGBT+ community” (the acronym was expanded to include “Queer”). A higher mean score indicates a higher sense of connectedness. When validated, this scale had excellent internal reliability (α = 0.81 [56]) and in this study (α = 0.98).

The Brief Condom Use Self-Efficacy Scale [43], with pronouns adjusted to be gender neutral (i.e., he/she > they), was used. This is a seven-item scale, measured on a five-point Likert scale (1—completely disagree, 5—completely agree). An example item is “I am sure that I would remember to use a condom although I have consumed alcohol or other drugs”. A higher score indicates higher perceived condom self-efficacy. Wider research has shown acceptable internal reliability (α = 0.71 [43]) as well as in this study (α = 0.70).

The PrEP Self-Efficacy Behavior Subscale was used [78]. This contains eight items such as “How difficult would it be for you to visit a doctor who can provide PrEP?”, measured on a four-point Likert scale (1—very hard to do, 4—very easy to do). A higher score indicates higher PrEP self-efficacy. Previous related research shows this to have good internal reliability (α = 0.87 [45]), as is the case in this study (α = 0.78).

The revised Sociosexual Orientation Inventory [79] captures three theoretically meaningful components of sociosexuality using nine items. Example items include “With how many partners have you had sex within the last 12 months?” (past behavioral experiences); “Sex without love is ok” (attitude towards uncommitted sex); and “In everyday life, how often do you have spontaneous sexual fantasies about having sex with someone you have just met?” (sociosexual desire). Items were measured on various nine-point Likert scales, and an amalgamated score was calculated. A higher mean score indicates higher sociosexuality. In a related study this had excellent internal reliability (α = 0.84 [80]), as is the case in this study (α = 0.86).

### 2.3. Statistical Analysis

All scale items were coded such that higher scores reflected higher levels of the construct being measured. Where applicable, reverse-worded items were recoded prior to analysis. Composite scores for each measure were calculated by averaging the relevant items, with higher mean scores indicating higher levels of the construct. Variables were analyzed in their original metric and were not standardized prior to the mediation analyses. Two mediation analyses were conducted using the PROCESS macro for SPSS (Version 4.2 [81]). In the first model, identity resilience was specified as the independent variable, internalized homonegativity specified as the mediator, and sociosexuality as the outcome variable. In the second model, LGBT+ connectedness was specified as the independent variable, internalized homonegativity again as the mediator, and sociosexuality as the outcome variable. In each analysis, the alternative predictor was included as a covariate to estimate the unique indirect effect of the focal independent variable. Condom self-efficacy and PrEP self-efficacy were also included as covariates due to their established associations with sexual decision-making. Indirect effects were estimated using 5000 bootstrap samples with 95% confidence intervals. Indirect effects were considered statistically significant when the bootstrapped confidence interval did not include zero.

Although causal mediation is established using longitudinal and/or experimental designs that permit empirical tests of temporal precedence, mediation-type models are also used with cross-sectional data to estimate theoretically specified indirect effects, provided that interpretation is appropriately bound to associational pathways rather than definitive causal processes [82]. Thus, cross-sectional mediation modeling is nevertheless widely used within psychological and public health research as an exploratory approach for examining potential mechanisms and informing future longitudinal or experimental investigations. Recent studies have adopted similar approaches to examine indirect pathways within cross-sectional datasets [83,84,85]. As such, we specified an *a priori* model and estimated indirect effects to test whether the observed pattern of associations was consistent with the hypothesized mechanism. The direction of paths in the current model was determined by theory rather than by the data alone (e.g., [47,63]).

## 3. Results

**Figure 2:**
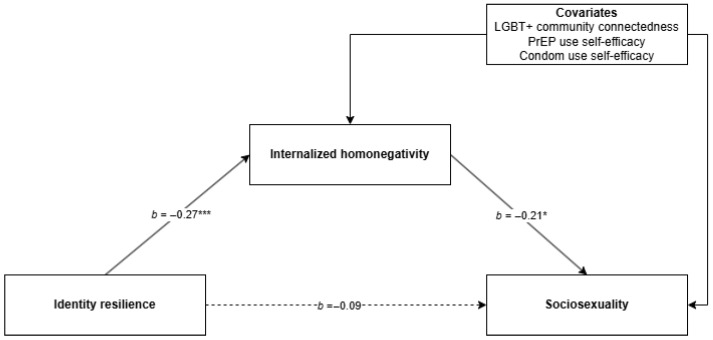
Mediation model predicting sociosexuality from identity resilience via internalized homonegativity. * Significant to *p* < 0.05, *** significant to *p* < 0.001.

**Figure 3:**
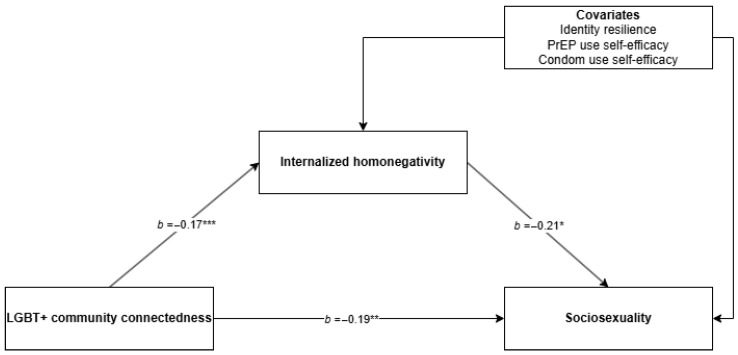
Mediation model predicting sociosexuality from LGBT+ connectedness via internalized homonegativity. * Significant to *p* < 0.05, ** singifianct to *p* < 0.01, *** significant to *p* < 0.001.

Two mediation analyses were conducted using PROCESS (Model 4 [86]) to examine whether internalized homonegativity mediated the associations between (a) identity resilience and sociosexuality, and (b) LGBT+ connectedness and sociosexuality (see Figure 2 and Figure 3). Bootstrapped confidence intervals were generated using 5000 resamples. No substantial violations of standard regression assumptions were identified (i.e., normality, linearity, homoscedasticity, and independence of residuals), and there were no issues of multicollinearity (i.e., all variance inflation factors < 1.5 [87]).

### 3.1. Identity Resilience, Internalized Homonegativity, and Sociosexuality

This model examined identity resilience as the focal independent variable, controlling for LGBT+ connectedness and the covariates. The overall model predicting sociosexuality was statistically significant, *F*(5, 506) = 13.15, *p* < 0.001, with *R*^2^ = 0.12, indicating that approximately 12% of the variance in sociosexuality was explained by identity resilience, internalized homonegativity, and the covariates. Identity resilience statistically significantly predicted lower internalized homonegativity (β = −0.27, *SE* = 0.06, *t* = −4.56, *p* < 0.001). Internalized homonegativity again significantly predicted sociosexuality (β = −0.21, *SE* = 0.10, *p* = 0.035). However, the direct effect of identity resilience on sociosexuality was not statistically significant (β = −0.09, *SE* = 0.14, *t* = −0.69, *p* = 0.49).

The indirect effect of identity resilience on sociosexuality via internalized homonegativity was statistically significant (β = 0.06, bootstrapped *SE* = 0.03, 95% CI [0.001, 0.13]). The direct effect of identity resilience on sociosexuality was not statistically significant once internalized homonegativity was included in the model, suggesting that the association between identity resilience and sociosexuality operated indirectly through internalized homonegativity in this sample.

### 3.2. LGBT+ Connectedness, Internalized Homonegativity, and Sociosexuality

The mediation model predicting sociosexuality from LGBT+ connectedness was statistically significant, *F*(5, 506) = 13.15, *p* < 0.001, with *R*^2^ = 0.12, indicating that approximately 12% of the variance in sociosexuality was explained by LGBT+ connectedness, internalized homonegativity, and the covariates. Internalized homonegativity significantly predicted sociosexuality (β = −0.21, *SE* = 0.10, *t* = −2.12, *p* = 0.035), such that higher internalized homonegativity was associated with less sociosexuality. LGBT+ connectedness was a significant positive predictor of sociosexuality (β = 0.19, *SE* = 0.06, *t* = 2.89, *p* = 0.004), indicating a significant direct effect.

LGBT+ connectedness also significantly predicted lower internalized homonegativity (β = −0.17, *SE* = 0.03, *t* = −6.01, *p* < 0.001). The indirect effect of LGBT+ connectedness on sociosexuality via internalized homonegativity was statistically significant (β = 0.04, bootstrapped *SE* = 0.02, 95% CI [0.002, 0.08]), indicating partial mediation. This suggests that LGBT+ connectedness was associated with more sociosexuality both indirectly through reduced internalized homonegativity and directly via other pathways.

### 3.3. Covariates

Across both models, condom self-efficacy and PrEP self-efficacy were included as covariates in the mediator and outcome equations. Condom self-efficacy was negatively associated with sociosexuality (β = −0.56, *SE* = 0.11, *t* = −5.11, *p* < 0.001), whereas PrEP self-efficacy was positively associated with sociosexuality (β = 0.64, *SE* = 0.14, *t* = 4.74, *p* < 0.001). The inclusion of these covariates ensured that observed associations between identity-based factors, internalized homonegativity, and sociosexuality were independent of sexual health self-efficacy and risk-management confidence.

## 4. Discussion

This study set out to investigate how adaptive self-schema, relational schema, and identity evaluation variables relate to sociosexuality among gay and bisexual men. We hypothesized that two adaptive schemas, namely identity resilience and LGBT+ community connectedness, would each be associated with higher sociosexuality, and that these links would be mediated by decreased internalized homonegativity. PrEP use self-efficacy and condom use self-efficacy were included as covariates to examine the effects of the adaptive schema and identity evaluation variables independently of one’s self-reported confidence to engage in safer sex practices.

Although no hypotheses were made regarding the associations between the covariates and sociosexuality, PrEP use self-efficacy was a positive correlate and condom use self-efficacy a negative correlate of sociosexuality. It is possible that people who feel more able to use PrEP in turn feel more empowered to engage in sexual self-expression (operationalized as sociosexuality), while those who rely exclusively on condoms may feel more constrained in their sexual self-expression. This hypothesis is broadly consistent with the findings of other studies [88,89].

Hypothesis 1, predicting a direct positive association between identity resilience and sociosexuality, was not supported. Identity resilience alone did not have a significant direct effect on sociosexuality once the effects of internalized homonegativity were accounted for. Identity resilience influenced sociosexuality only indirectly via lower internalized homonegativity. Hypothesis 2, which posited a positive direct link between LGBT+ community connectedness and sociosexuality, conversely, was supported. Hypothesis 3, concerning mediation, was partially supported: internalized homonegativity significantly mediated the effects of both identity resilience and LGBT+ community connectedness on sociosexuality. For identity resilience, the direct association with sociosexuality was not statistically significant once internalized homonegativity was included in the model, suggesting that the relationship was accounted for indirectly via internalized homonegativity. Regarding LGBT+ community connectedness, the mediation was partial and a direct pathway remained alongside the indirect effect.

Findings contribute to a growing body of literature re-framing sociosexuality in sexual minority men as a potentially positive aspect of identity and social life [90]. Past research with predominantly heterosexual samples has consistently linked “unrestricted” sociosexuality to maladaptive characteristics, such as higher levels of trait narcissism and psychopathy, aggression, impulsivity, and insecure attachment [91,92,93,94,95]. In gay and bisexual men, much of the earlier work has similarly viewed high sociosexuality through a pathological or risk lens, focusing on the increased likelihood of STI/HIV acquisition [9,96,97].

In contrast, our study aligns with emerging research that shows that sociosexuality among gay and bisexual men can be a form of identity expression and community connection. This would explain the positive direct and indirect effects of LGBT+ community belonging and identity resilience, respectively. Wilkinson et al. found that casual sexual encounters often paved the way for friendships and social bonds among gay men, indicating that seeking sex can be intertwined with seeking connection [10]. Furthermore, Jaspal and Papaloukas reported that sex in communal spaces, such as gay saunas, provided not only physical pleasure but also a sense of belonging and authenticity, helping to alleviate feelings of isolation and loneliness [14]. In her ethnographic study of gay men in Los Angeles, Stacey found that engagement in recreational sex served “as a cultural resource for constructing creative ‘families of choice’”, enabling gay men to form intimate attachments that cut across various other identity characteristics, such as generation, social class, and race [32].

These perspectives frame sociosexuality as a prosocial cognitive, affective, and behavioral variable, that is, as a way to affirm one’s sexual identity, challenge heteronormative norms about monogamy, and cultivate community ties. This study supports this positive framing, with higher levels of sociosexuality being associated with healthier identity profiles (i.e., higher identity resilience and lower internalized homonegativity) and strong community identification. This suggests that, rather than stemming from appraisal or minimization of “risk”, sociosexuality in this context is associated with personal and social wellbeing. This is especially important, given that the observed associations remained significant even when accounting for PrEP use self-efficacy and condom use self-efficacy, indicating that sociosexuality was linked to self-schema, relational schema, and identity evaluation variables over and above individual differences in sexual health risk management.

More generally, these results are consistent with emerging evidence concerning the significance of relational and self-schemas, as well as identity evaluation factors, in psychological wellbeing among gay and bisexual men. For instance, Jaspal found that adaptive relational and self-schemas were associated with positive identity changes that in turn increased the likelihood of coming out as gay (i.e., sexual identity enactment) [68]. Similarly, Jaspal et al. found that adaptive self-schemas and relational schemas were associated with the derivation of sexual satisfaction among gay and bisexual men [51]. In short, how gay and bisexual men think about their own identity and their level of connectedness with others appears to be associated with how they enact their sexual identity, namely their degree of sociosexuality. Although the data are cross-sectional correlational, it is legitimate to conceptualize identity resilience (a self-schema) and community connectedness (a relational schema) as antecedents to lower internalized homonegativity (a mutable identity evaluation construct), since this is consistent with the findings of previous studies [53,97].

The results support a theoretical view of sociosexuality as an important aspect of at least some gay and bisexual men’s identities and wellbeing. Rather than viewing a high number of partners or openness to uncommitted sex as mere indicators of risk or deviance [98,99], our study suggests they may, under some circumstances, be indicators of positive adjustment, signifying that an individual has achieved a level of self-acceptance and community integration that in turn allows them to express their sexuality freely. These findings should encourage researchers and clinicians to adopt a sex-positive framework when considering sociosexuality in gay and bisexual men. For instance, sexual health programs can emphasize that enjoying casual sex is a valid expression of one’s identity, especially when accompanied by measures that ensure safety and consent [99,100].

Furthermore, the findings highlight potential targets for interventions aiming to improve gay and bisexual men’s wellbeing. Reducing internalized homonegativity is, and should remain, a key goal, given the broader health and wellbeing benefits of doing so [66]. Interventions (e.g., counseling, support groups, or community programs) that help gay and bisexual men challenge and overcome negative beliefs about their identity could not only improve mental health but also allow individuals to engage more comfortably in desired sexual relationships. For instance, Ross et al. [101] targeted internalized homonegativity in a cohort of 55 sexual minority individuals using modified cognitive behavioral therapy in group-based sessions, thereby alleviating depression. Similarly, Lin and Israel [102] found that their online intervention consisting of three modules was successful in reducing internalized homonegativity in gay and bisexual men. The same intervention was refined and replicated in a subsequent study [67]. Our findings build on this evidence and suggest that interventions that boost feelings of identity resilience and LGBT+ community connectedness may also be effective in reducing internalized homonegativity, thus facilitating sociosexuality.

This research has several limitations that warrant consideration in future studies. First, the study’s cross-sectional design limits our ability to draw causal conclusions. This study focuses only on associations between these variables. Longitudinal and experimental data would help clarify the directionality of these effects and provide further evidence of mediation. Second, the study potentially suffers from sampling bias. Participants were recruited using convenience sampling and, thus, the generalizability of the findings is limited. This approach is likely to skew the sample toward gay and bisexual men who are already relatively open about their sexuality and more connected to LGBT+ communities. Consequently, gay and bisexual men who are more socially isolated or less open about their sexuality may be underrepresented in the sample and, incidentally, it is these men who are more susceptible to internalized homonegativity. It is important to replicate this study using representative samples of gay and bisexual men. Third, these findings cannot be assumed to generalize to other sexual and gender minority populations, such as lesbian, transgender, and gender-diverse individuals, whose social contexts and identity processes may differ. For instance, transgender men who identify as gay or bisexual may face additional stressors relating to their gender identity that operate as cumulative stressors that may affect their degree of sociosexuality [72,73]. Future research should therefore examine these relationships across the broader spectrum of LGBT+ identities to establish the wider applicability of the model. Finally, although our model explained a statistically significant portion of variance in sociosexuality, the observed effect sizes were modest. This suggests that many other factors contribute to one’s degree of sociosexuality. Possible confounds include personality traits (e.g., extraversion or openness to experience), relationship status, age, and generational cohort.

## 5. Conclusions

In conclusion, this study advances our understanding of sociosexuality among gay and bisexual men by situating it within the context of identity resilience, community belonging, and internalized homonegativity. Findings indicate that, rather than reflecting maladjustment or a propensity to take risks, openness to casual sex may, under some circumstances, represent the enactment of a confident and socially supported sexual identity. Gay and bisexual men with more resilient identities and stronger connections to the LGBT+ community reported more unrestricted sociosexual attitudes and behaviors, particularly when internalized homonegativity was low. As such, comfort with casual sex appears closely tied to self-acceptance and community affirmation.

These findings support a more affirmative conceptualization of sociosexuality—one that recognizes the significance of safety and prevention while also acknowledging the psychological and social benefits of sexual exploration in the context of contemporary biomedical advances such as PrEP. By supporting the development of identity resilience and community connectedness while addressing internalized homonegativity in clients, practitioners may help create the conditions in which sociosexuality can enhance, rather than undermine, overall wellbeing. More broadly, the findings support a holistic conceptualization of sexual health that incorporates identity affirmation, pleasure, and connection.

## Data Availability

The datasets presented in this article are not readily available because of ethical restrictions. Data can be obtained through reasonable request from the corresponding author.

## Abbreviations

The following abbreviations are used in this manuscript:

HIV	Human immunodeficiency virus
STI	Sexually transmitted infections
PrEP	Pre-exposure prophylaxis
U = U	Undetectable = Untransmittable
